# Engineering with care: safety assessment platforms for CRISPR-modified natural killer cells

**DOI:** 10.3389/fimmu.2025.1711414

**Published:** 2025-12-05

**Authors:** Alieh Fazeli, Evelyn Ullrich, Toni Cathomen, Tobias Bexte

**Affiliations:** 1 Institute for Transfusion Medicine and Gene Therapy, Medical Center - University of Freiburg, Freiburg, Germany; 2 PhD Program, Faculty of Biology, University of Freiburg, Freiburg, Germany; 3 Department of Pediatrics, Experimental Immunology and Cell Therapy, Goethe University Frankfurt, Frankfurt am Main, Germany; 4 Frankfurt Cancer Institute, Goethe University Frankfurt, Frankfurt am Main, Germany; 5 German Cancer Consortium (DKTK) partner site Frankfurt/Mainz, Frankfurt am Main, Germany; 6 Center for Cell and Gene Therapy, Medical Center - University of Freiburg, Freiburg, Germany; 7 German Cancer Consortium (DKTK) partner site Freiburg, Freiburg, Germany; 8 Faculty of Medicine, University of Freiburg, Freiburg, Germany; 9 German Red Cross Blood Service Baden-Württemberg - Hessen, Institute for Transfusion Medicine and Immunohematology, Frankfurt am Main, Germany; 10 Berlin Center for Advanced Therapies, Charité – Universitätsmedizin Berlin, Berlin, Germany

**Keywords:** CRISPR, NK cells, safety assessment, off-target, allogenic, gene-editing, cell therapy

## Abstract

CRISPR-based gene editing has become a transformative tool to enhance immune cell therapies. In particular, engineering natural killer (NK) cells with CRISPR/Cas systems has gained traction due to their ability to mediate strong anti-tumor responses in an MHC-unrestricted, non-alloreactive manner. Early trials show the feasibility and safety of allogeneic NK cells, paving the way as scalable “off-the-shelf” products. CRISPR/Cas9 edits genomes by inducing DNA double-strand breaks (DSBs), mainly repaired through non-homologous end joining (NHEJ) or homology-directed repair (HDR). While effective, CRISPR carries risks of off-target (OT) activity that may disrupt essential genes, cause chromosomal rearrangements, or trigger oncogenic changes - posing threats to product integrity and patient safety. These concerns intensify with multiplex editing, where multiple loci are modified to improve function, persistence, and immune evasion. Since unmodified NK cells are typically short-lived, many clinical-stage products are engineered to express IL-15 or related constructs, extending their half-life and amplifying risks associated with unintended changes. This underscores the urgent need for robust safety assessments. In this review, we summarize the current landscape of safety assessment platforms for evaluating gene edited NK cells. We highlight predictive in silico tools, biochemical *in vitro* assays, and emerging cell-based detection systems to identify and quantify CRISPR-induced OT events. Particular attention is given to their suitability, limitations, and practical use in primary NK cells and multiplex editing strategies. Our aim is to support the design of safe, effective editing workflows for NK cell therapies - ensuring rigor as the field advances rapidly toward clinical application.

## Introduction

The bacterial clustered regularly interspaced short palindromic repeats (CRISPR)-CRISPR-associated protein 9 (Cas9) was engineered for RNA-programmable DNA cutting the first time in 2012 (1, 2). Today this system has undergone several cycles of optimization and been frequently adapted for precise editing in several human cells including therapies based on T and NK cells (3). The concept is simple: target gene recognition by the guide RNA (gRNA), which leads to a double stranded break (DSB) introduced by the Cas9 protein. The resulting DSB is then repaired by nonhomologous end-joining (NHEJ) or homology-directed repairs (HDR). In context of primary immune cells, delivery of a gRNA/Cas9 ribonucleoprotein (RNP) complex was identified as the most stable version for primary cell editing and is therefore most frequently used and entered multiple clinical trials (4–6). Therefore, CRISPR applications have become a powerful tool to enhance the therapeutic efficacy of primary immune cell therapies (7–10).

In recent years the application of Natural Killer (NK) cells came to the focus as an emerging promising approach for a particularly safe cellular immunotherapy for cancer (11–13). Their intrinsic functionality and their capacity to recognize infected or transformed cells that lack major histocompatibility complexes (MHC), position them as a promising strategy for targeted cancer treatment (14). In addition, the lack of rearranged antigen receptors enables their use in allogeneic, “off-the-shelf” applications. In this regard, early clinical data indicate that allogeneic NK cell applications show a very favorable safety profile compared to chimeric-antigen-receptor (CAR)-T cells (15, 16). Incorporating a CAR into NK cells can further improve their tumor-targeting capacity, and early-phase clinical trials have demonstrated encouraging responses with CAR−NK cell therapies (12, 15–17). Today, there are more than 120 registered CAR-NK cell trials, for hematological cancers, solid tumor and autoimmune and infectious diseases (18). All of them do not show Graft-versus-Host Disease (GvHD), neurotoxicity (ICANS) and almost no cytokine-release-syndrome (CRS) (12, 18).

Although the patient’s response rate is promising, the NK cells functionality can be impaired by inhibitory receptors and negative regulation by the tumor microenvironment (TME), restricting their anti-tumor capacity (17, 19, 20). In addition, NK cells are typically short-lived and their killing activity is balanced by different activating and inhibitory receptors, which was shown to limit their clinical benefit, also of CAR-NK cells (11, 20). These biological constraints highlight the need and enormous potential for genetic engineering strategies to enhance NK cell function and persistence while maintaining their favorable safety profile. The recent developments of CRISPR applications opens the advertisements for a broad potential for precise engineering tools as a powerful approach to boost the therapeutic window of NK cell therapy as a safe cell therapy product (11, 12, 19, 21, 22). Therefore, the rationale for genome editing in NK cells stems from the need to overcome the key limitations without hurting NK cell therapy’s impeccable safety records.

Suboptimal activation and cytotoxicity can result from inhibitory signaling and insufficient activating receptor engagement. To address this, CRISPR has been used to disrupt or enhance the expression of genes involved in receptor-mediated recognition, such as *KLRC1* (encoding NKG2A), *SIGLEC7* (sialic acid-binding Ig-like lectin 7), *CD96*, *KLRK1* (killer cell lectin-like receptor subfamily K, member 1; also known as NKG2D), *NCR1* (natural cytotoxicity triggering receptor 1), *NKp46* (natural killer cell protein 46), and *NCAM1* (encoding CD56) (23–32). Furthermore, editing of immune checkpoint regulators, including *CISH* (cytokine-inducible SH2-containing protein), *PD1* (programmed cell death protein 1), *TIGIT* (T cell immunoreceptor with Ig and ITIM domains), and *A2AR* (adenosine A2A receptor), has been shown to restore anti-tumor activity and improve *in vivo* persistence (33–37). To enhance signaling downstream of activating receptors, genes such as *SYK* (spleen tyrosine kinase), *CD247* (encoding CD3 zeta chain), *FCGR1A* (Fc receptor gamma chain), and *CBLB* (Casitas B-lineage lymphoma proto-oncogene B) have been modified to amplify NK cell activation and cytokine production (36, 38). In addition, metabolic and cell cycle regulators such as *TGFBR2* (Transforming Growth Factor Beta Receptor II) and *HPRT1* (Hypoxanthine Phosphoribosyltransferase 1) have been targeted to enhance NK cell function and fitness. Finally, CRISPR-based genetic enhancement of targeting specificity and tracking has been achieved recently also through the knock-in of CAR constructs and reporter genes (e.g., CD19, CD33, GD2, EGFP, mCherry) (23, 33, 39–43).

Overall and most importantly, the limited *in vivo* persistence and proliferative capacity of NK cells can reduce the durability of functional NK cell responses for the patients (15). To address this, the field introduced NK cells specific cytokine support elements such as IL-15 to improve survival and expansion kinetics (15, 16, 37, 44–48). Almost all of the recent CAR-NK cells studies include IL-15 elements and confirmed an improved response by longer persisting CAR-NK cells without cytokine related toxicity (15, 16). However, other pre-clinical studies with genetically extended *in vivo* half-life describe systemic adverse effects *in vivo* in xenograft models by IL-15-armored NK cells (37, 49, 50).

Even though CRISPR/Cas is a very efficient and precise gene editing tool it can lead to DNA cuts at sites other than the intended target site, primarily when the mismatch-tolerating CRISPR/Cas complex mediates binding to DNA sequences that resemble the target site closely. Furthermore, unintended structural variants, such as large on-target site deletions, chromosomal rearrangements, or chromosomal instability have been reported (51–53). Such unintended editing outcomes can – in principle – disrupt vital genes, activate/inactivate undesired genes, cause genomic instability or even oncogenic transformation, ultimately endangering patient safety (52–58). To address some of these limitations, new CRISPR/Cas types like base editors (BE) and prime editors (PE) open new potential for introducing point-mutations and thereby gain- or loss-of-function without depending on DSBs or on an HDR template for precise repair (59). However, the application in NK cells is still limited (35, 37). One study recently reported the first CAR transgene integration in combination with multiplex BE (using up to six guides) in primary NK cells (37). The multiplex edited IL15-expressing CAR-NK cells showed enhanced in−vitro potency. However, although the xenograft mice models confirmed an increased antitumor activity, they also revealed major organ toxicity in the multiplex edited CAR-IL15 NK cell treatment group, limiting the overall survival (37, 60). To assess genomic safety, the authors analyzed editing at the top computationally predicted OT sites using rhAmpSeq (RNase H2-dependent multiplexed PCR Sequencing). While they did not identify OT effects and negligible rates of translocations between on-target sites using this approach, it should be noted that only genome-wide techniques ensure sensitive detection of OT activity and therefore safe clinical translation (52, 61, 62).

**Figure 1:**
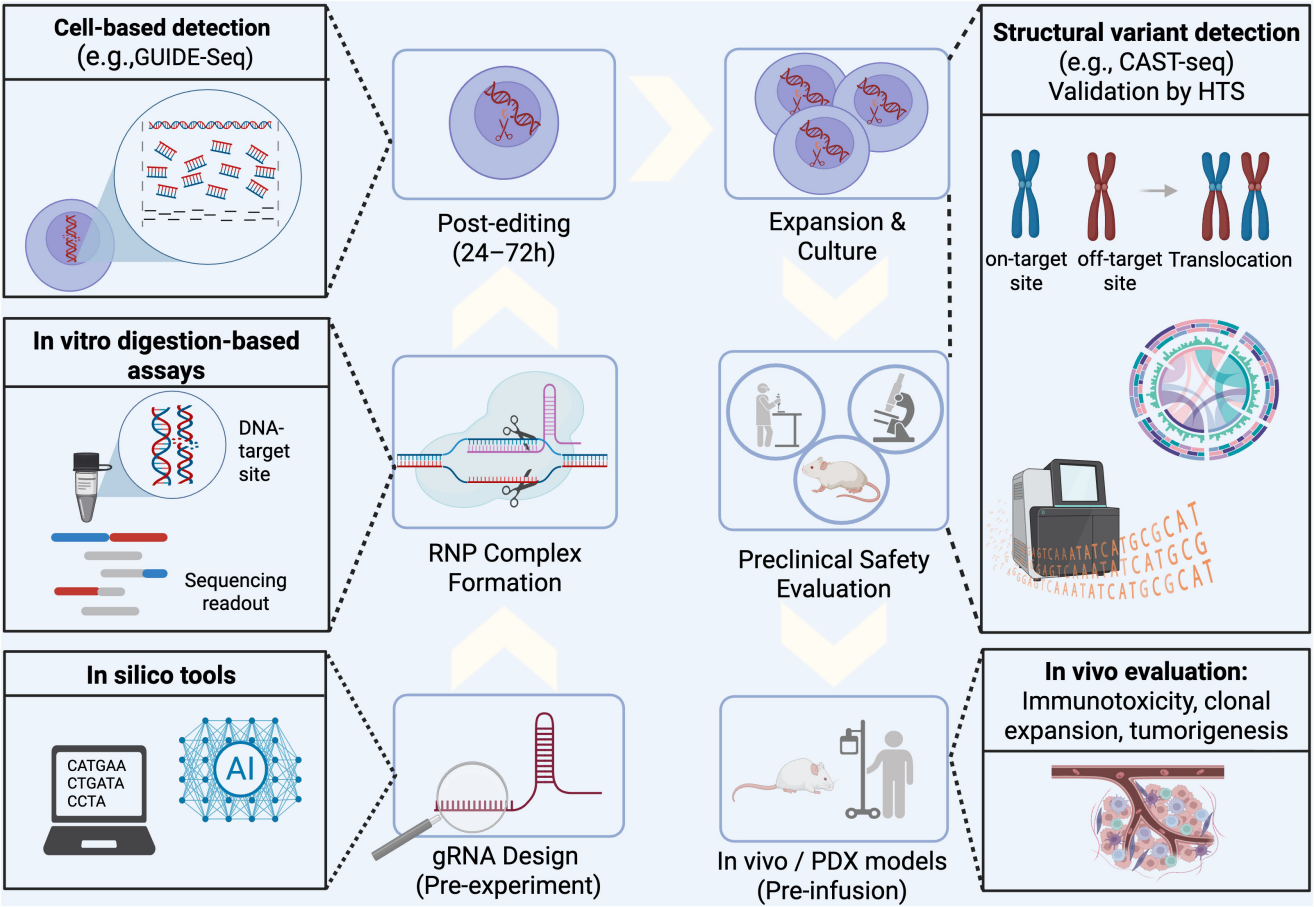
Off-target detection strategies across the CRISPR-NK cell editing workflow. Illustration of key stages in NK cell genome editing and corresponding safety assessment tools. *In silico* tools (e.g., CRISPOR, DeepCRISPR) assist with gRNA design before editing. *In vitro* digestion-based methods (e.g., CIRCLE-seq, SITE-seq, Digenome-seq) find cleavage sites in cell-free genomic DNA. Cell-based assay (e.g., GUIDE-seq, DISCOVER-seq) detects DSB-associated off-targets under chromatin context. Structural variants are captured post-expansion using CAST-seq and validated by high-throughput sequencing (HTS). *In vivo* post-infusion studies assess clonal expansion and tumorigenesis. Created with Biorender.com. AI, Artificial intelligence; DSB, Double-strand break; gRNA, Guide RNA; HTS, High-throughput sequencing; RNP, Ribonucleoprotein; SNV, Single-nucleotide variant; PDX, Patient-derived xenograft model.

In the last years several detection techniques have been developed to profile and mitigate unintended edits, so minimizing OT activity in therapeutic genome editing (63, 64). These include in silico prediction tools, *in vitro* biochemical tests, and cell-based methods; each of them have their unique benefits and drawbacks (**Figure 1**). From a translational point of view, it is important to note that regulatory agencies increasingly require orthogonal OT validation, i.e. a combination of in silico predictions, *in vitro* cleavage assays, and cell-based detection platforms to ensure comprehensive detection of potential OT events (65, 66). The most suitable combination of assays may depend on the cell type, the editing tool, and the intended use of the cell therapy. In particular for multiplex editing strategies in NK cells (19), an urgent need for rigorous and comprehensive safety analysis is necessary to not hurting NK cell therapy’s impeccable safety records in the early clinical trials.

## *In silico* prediction tools

In silico tools serve as an essential initial step by computationally predicting potential OT sites based on gRNA sequence similarity across the genome (**Figure 1**). Traditional tools such as CRISPOR, CCTop (CRISPR/Cas9 Target online predictor), and COSMID (CRISPR OT Sites with Mismatches, Insertions, and Deletions) use sequence alignment and mismatch tolerance models derived from Cas9 activity data to generate ranked lists of potential OT sites (67–70). More recently, deep learning–based tools such as DeepCRISPR, DeepCBE, DeepABE and DeepBE have been developed to predict the efficiencies of BEs and their OT propensities (71, 72). These tools are especially useful in the early design phase of CRISPR editing experiments. Although in silico tools are fast and cost-efficient, their predictions require empirical validation as chromatin accessibility and DNA repair pathway choice have a substantial impact on editing outcomes that cannot be predicted yet (67, 71).

Before performing CRISPR/Cas9 editing in primary NK cells or NK-92 lines, authors in a number of NK cell CRISPR studies first designed gRNAs using in silico tools like Benchling, giving priority to high predicted on-target scores and minimal OT potential (23, 25, 33, 36). In the past years, some studies used next-generation high-throughput sequencing (HTS) to confirm a subset of in silico predicted OTs, while others reported no OT analysis at all (**Table 1**), possibly as a result of financial constraints, technological limitations, or presumptions regarding the CRISPR/Cas9 system’s high specificity (30, 40, 73, 74). The absence of such analysis raises concerns about the completeness of safety assessments, especially in therapeutic settings.

**Table 1:** Off-target detection methods applied in CRISPR-edited NK cells.

Detection method	Advantage	Disadvantage	Study	NK cell type	Key off-target insight
*In silico*	- Fast and cost-effective - Useful for initial gRNA design	- Prediction only; no confirmation of actual cleavage - Misses many true-positives (i.e. low sensitivity) - Reports many false-positives (i.e. low specificity)	(23)	NK-92 cell line	Benchling predicted OT sites; no OT activity at top predicted OT sites detectedby HTS
			(36)	PBMC-derived NK & NK-92 cell line	Designed with Synthego’s CRISPR Design tool (in silico ranking); no empirical validation
*In vitro*: CHANGE-seq	- High-throughput - Captures broad OT profile	- Performed on naked genomic DNA - Reports many false-positives (i.e. low specificity)	(42)	PBMC-derived NK	Minimal OT activity at some nominated OT sites detected by HTS and long-read WGS
*In cellula*: GUIDE-seq	- High sensitivity - Detects OT sites in living cells	- Requires integration of ssODN tag - Inefficient in NK cells due to toxicity of dsODN - Performed in surrogate cell line that does not reflect epigenetic state of NK cells	(82)	PBMC-derived NK	GUIDE-seq in HEK293; validated by HTS in NK cells; No OT activity at some nominated OT sites detected
			(34)	Cord blood-derived CAR-NK cells	GUIDE−seq in HEK293; validated by HTS in CAR−NK cells; Cas9 showed low OT frequency with single or combined gRNAs; HiFi Cas9 reduced OT to <0.5%.
No method used			(83)	PBMC-derived NK cells	
			(35)	PBMC-derived NK cells	

Summary of *in silico*, *in vitro*, and *in cellular* platforms used in published studies to evaluate CRISPR-specificity in NK cells. OT, Off-target; HTS, High-throughput sequencing; WGS, Whole-genome sequencing; dsODN, Double-stranded oligodeoxynucleotide; PBMC, Peripheral blood mononuclear cells; CAR, Chimeric antigen receptor.

## *In vitro* detection assays

For *in vitro* detection assays, purified genomic DNA is treated with nucleases such as CRISPR/Cas9 in a cell-free environment using biochemical techniques like CIRCLE-Seq, SITE-Seq, CHANGE-Seq and Digenome-Seq (75–78). HTS is then used to map cleavage sites (**Figure 1**). While disregarding chromatin context or cellular repair, these assays offer objective, sensitive mapping of DNA breaks (75–77). For instance, OT sites with high or low cleavage frequencies can be found using SITE-Seq (Selective Enrichment and Identification of Tagged Genomic DNA Ends by Sequencing), which works by tagging DSBs with biotinylated adapters, enriching these fragments, and mapping them via high-throughput sequencing (76). CIRCLE-Seq (Circularization for *in vitro* reporting of cleavage effects by sequencing) and its successor CHANGE-seq (Circularization for High-Throughput Analysis of Nuclease Genome-wide Effects by sequencing) use circularized fragments of purified genomic DNA, which are then incubated with CRISPR/Cas9 to identify cleaved sites (75, 78, 79). Digenome-Seq uses whole-genome sequencing to identify cleavage sites based on sequence break points after treating naked genomic DNA with Cas9 *in vitro* (80).

However, because these assays are conducted outside of the cellular environment, they might cut DNA sequences where chromatin compaction or epigenetic regulation prevent Cas9 from cleaving in cells (75, 81), often leading to an overestimation of OT sites. The sensitivity and specificity of any of these assays have not yet been evaluated in the context of NK cells, despite the fact that they are frequently utilized for *in vitro* OT profiling (**Table 1**).

## Cell-based detection approaches

Cell-based methods such as GUIDE-seq and DISCOVER-seq detect OT cleavage sites within living cells, offering valuable insight into genome editing specificity under physiologically relevant conditions (**Figure 1**) (80, 84–86), especially if performed in the clinically relevant cell type. GUIDE-seq (Genome-wide Unbiased Identification of DSBs Enabled by Sequencing) introduces double-stranded oligodeoxynucleotides (dsODNs) along with the CRISPR/Cas9 nucleases. As these dsODNs will be incorporated at DNA double-strand breaks (DSBs), GUIDE-seq allows for genome-wide mapping of Cas9-induced OT sites (80). However, dsODNs can be toxic to primary cells, including NK cells, which makes GUIDE-seq less useful in cell types that are important for clinical use. DISCOVER-seq (Discovery of *In Situ* Cas OTs and Verification by End-Ligation and Repair Sequencing) is based on chromatin immunoprecipitation followed by sequencing (ChIP-seq) targeted to the DNA repair factor MRE11, which is rapidly recruited to DSBs. With the help of this technique, Cas9-induced DNA breaks in living cells can be tracked genome-wide, offering dynamic insights into editing activity in a chromatin-dependent setting (84). While this method offers chromatin-aware OT detection, its application in NK cells remains limited. One publication demonstrating the application of cell-based detection assay for primary NK cells comes from a study targeting the *CD38* locus (55). In order to find potential OT sites, GUIDE-seq was first performed in HEK293 cells using a *CD38*-targeted gRNA. These nominated sites were then evaluated in CRISPR/Cas9-edited primary NK cells using rhAmpSeq (82). The resulting data revealed a high on-target editing efficiency and no detectable OT editing (82).

As summarized in **Table 1**, most studies that applied GUIDE-seq in the context of NK cell engineering, including the example discussed above, performed the initial GUIDE-seq screen in surrogate HEK293 cell lines, only the nominated sites were validated in primary NK cells (34, 82). This leaves open the possibility that additional OT events unique to NK cells may have gone undetected. Therefore, for clinical applications, safety evaluations of NK cell-based therapies must be carried out directly in NK cells rather than extrapolated from surrogate cell lines because OT profiles are impacted by cell-type-specific factors like chromatin accessibility, DNA repair mechanisms, and delivery efficiency (87).

Apart from cleavage-based tests, CAST-Seq (Chromosomal Aberration analysis by Single Targeted linker-mediated PCR followed by sequencing) makes it possible to identify structural changes like translocations and large deletions and inversions after CRISPR/Cas9 editing, especially in therapeutic applications where chromosomal stability is crucial (88–93). Notably, CAST-Seq can also nominate potential OT sites that can be further validated by HTS, a strategy that has been employed in several recent studies (88–90, 94). Another advantage of this method is, in contrast to many other genome-wide assays, its compatibility with low cell input, making it especially useful in settings where only limited numbers of edited cells or genomic DNA are available, such as for primary NK cells in early-stage clinical manufacturing or cells isolated from patients or preclinical animal models (**Figure 1**). Despite not having been applied to NK cells in published studies yet, CAST-Seq is a promising tool for evaluating genomic integrity in next-generation NK-based therapies (**Table 1**).

As mentioned above, while researchers have applied various OT detection methods in edited (CAR) T cells and hematopoietic stem/progenitor cells (HSPCs) (88, 95–97). They have been applied only to a limited extent to NK cells (**Table 1**). The application of comprehensive, genome-wide OT detection techniques to NK cells is however vital in order to assure the safe clinical application of this cell type. Given the therapeutic potential of genome-edited NK cells, particularly as readily accessible off-the-shelf cell therapy, applying next-generation OT detection platforms to primary NK cells is imperative (98). Combining optimized in silico prediction pipelines with empirical, genome-wide methods in a standardized processes will ultimately improve the safety profile of this emerging therapy (99).

## Pre-clinical *in vivo* models for immunotoxicity

An additional dimension for pre-clinical safety evaluation of engineered NK cells lies in the appropriate choice and use of animal or alternative *in vivo* or *in vivo*-like models, including zebrafish xenografts, chorioallantois membrane (CAM) assays, and micro physiological organ-on-chip systems.

In addition to classical animal and humanized mouse models, several alternative *in vivo*-like systems are emerging that could expand the safety-testing toolbox for engineered NK cells. The CAM assay, long used for tumor xenografts and angiogenesis studies, provides a rapid and cost-efficient intermediate model between *in vitro* and murine experiments. While it has already been applied for CAR T-cell efficacy testing, its use with NK cells remains largely unexplored and may offer a convenient platform for short-term assessment of cytotoxicity and tissue interactions (100). Similarly, organ-on-chip and 3D perfused tissue constructs recreate vascularized, microfluidic environments that enable real-time monitoring of NK-cell migration, infiltration, and tumor killing under near-physiological flow conditions. These systems can help dissect cell-intrinsic behavior and immune-microenvironment interactions before moving to full animal studies (101). To limited extent, alternative *in vivo* systems such as zebrafish larval xenografts are used as rapid, cost-effective platforms to visualize NK-cell cytotoxicity and tissue interactions before advancing to murine studies (102). These *in vivo* platforms can permit assessment of persistence, biodistribution, cytokine release, off-target toxicity and immune-host relationships in conditions closer to the human situation, yet they also have limitations (103, 104).

So far human-cell derived NK products are typically tested in immunodeficient mouse (or humanized mouse) xenograft models, allowing evaluation of both anti-tumor efficacy and safety (e.g., in murine lymphoma or solid tumor models) (**Figure 1**) (103, 105). In this regard, one study reported about the cause of severe toxicity in glioblastoma xenograft mouse models after different dose treatments with NK cells engineered to express IL-15 (weight loss and extensive NK cells infiltration) (49). Interestingly, it was not observed with transgenic IL-21 in the same *in vivo* model (49). Another study reported that armoring NK cells with secreted IL-15 enhanced the anti-AML functionality *in vitro* significantly, but in the *in vivo* model, the constitutive IL-15 expressing NK cells showed improved persistency but also revealed unexpected lethal toxicity (50). These highlights the need for such rigors pre-clinical *in vivo* platforms. However, translation from *in vivo* findings to humans also shows limitations, as to date none of the ongoing clinical trials evaluating CD19-CAR NK cells co-expressing cytokines like IL-15 have reported such adverse effects (15, 16). Currently, xenograft mouse models are among the most well-studied models, but they also not fully recapitulate the human immune microenvironment and do not reflect the entire human immunology complexity (106, 107).

Therefore, standardized reporting of safety endpoints (dose, persistence, cytokine/chemokine panels, histopathology of host organs, biodistribution) and use of more physiologically relevant models (e.g., humanized immune system mice, canine models) are thus recommended (103). Incorporating these pre-clinical *in vivo* and *in vivo-*like platforms into the safety assessment pipeline for NK cell therapies could therefore reduce risk, improve translational predictiveness, and ultimately increase the likelihood of clinical success (**Figure 1**).

## Clinical application of CRISPR-edited NK cells

First genome-edited NK cell therapies are now in clinical or advanced preclinical development. Their current goals are to make them better at fighting tumors, lasting longer, and if quipped with CARs, also to avoid them from targeting themselves (fratricide). In this regard, FT538 (Fate Therapeutics) is one of the most advanced candidates. The iPSC-derived NK cell product is based on multiplexed genome editing using CRISPR/Cas9. It includes knocking out *CD38* to stop fratricide and adding a high-affinity, non-cleavable CD16 receptor (hnCD16) and an IL-15 receptor fusion (IL15RF) to help improve their *in vivo* persistence and boost their specific antibody-dependent cellular cytotoxicity (ADCC) (108). Although they entered first clinical trials, there was no detailed genome-wide OT profiling reported. However, by choosing single-cell iPSC clones based on karyotype analysis and targeted sequencing to make sure that there were no unintended genomic changes or integrations the risk of OT effects was lowered (108). Another candidate, FT596 that expresses a CD19-targeted CAR to eliminate hematologic malignancies, is also made from genetically modified iPSCs harboring hnCD16 and IL15RF (109). Interestingly, there is no public reports of OT analysis methods for this product so far (109).

## Pitfalls of today’s safety assessments: homologous receptor gene families and chromosomal instability

Editing at loci with high sequence homology to pseudogenes or paralogs can lead to misinterpretation of on-target activity and an underestimation of OT risks. An example is the locus coding for neutrophil cytosolic factor 1 (*NCF1*), a gene linked to chronic granulomatous disease. *NCF1* is flanked by two closely related pseudogenes with nearly identical sequences. Not surprisingly, it has been shown that CRISPR-editing of *NCF1* affects these pseudogenes, leading to large chromosomal rearrangements spanning several megabases (93). Furthermore, OT effects can be mistaken for successful on-target editing unless validated with allele-specific deep sequencing approaches (63). These events not only obscure functional interpretation but also pose a risk for chromosomal stability. Similar challenges occur when editing highly homologous NK cell receptor gene families, such as the NKG2 familiy (*KLRC1–4*), killer-cell immunoglobulin-like receptor members (*KIR2DS*, *KIR3DS*, *KIR2DL*, *KIR3DL*), natural cytotoxicity receptors (*NCR1–3*), *SIGLEC*, C-type lectin-like receptors *(KLRK1*, *KLRD1*, *KLRI1/2*), and leukocyte immunoglobulin-like receptors (*LILRB1*, *LILRB2*). Most of these gene families encode both activating and inhibitory receptors that share >85% sequence similarity in key exons but have distinct roles in NK cell regulation. Several members, including *KLRC1/NKG2A, KLRK1/NKG2D, KIR2DL, NCR1, SIGLEC7, SIGLEC9, and LILRB1*, have been targeted with CRISPR/Cas9 in primary NK cells or NK-92 lines to enhance cytotoxicity or study receptor function (11, 24, 29–32, 110). While one NKG2A study, using deep sequencing, reported minimal OT activity in CRISPR-modified knock-in NK cells integrating a GD2-CAR into the *KLRC1* locus, most other reports did not assess off-target effects, leaving open the possibility of inadvertently altering related family members, with potential consequences for NK cell phenotype and function (42). In this regard, CAST-Seq provides a valuable strategy to detect structural variants such as large deletions or translocations that may arise from editing genes in gene clusters, providing an additional safety layer for clinical-grade genome editing (93).

While unintentional cuts in oncogenes or tumor suppressors pose a well-known safety concern (52), chromosomal translocations between loci that are targeted at the same time (111), as well as the disruption of noncoding regulatory regions, such as enhancers and long non-coding RNAs (lncRNAs), may pose additional safety risks and could disrupt important immune functions without even changing the coding sequence (112). Because of these vulnerabilities, safety tests for NK cell editing should not only look for point mutations or small insertions/deletions, but must use methods to identify complex genomic aberrations, and take into account cell type-specific factors that affect the frequency, the nature and the consequences of OT editing outcomes (113).

## Future perspectives of today’s safety assessments

The rapid evolution of new editing technologies like BE offers an advantage over traditional CRISPR/Cas9 for multiplex gene editing, as they avoid the simultaneous induction of multiple DSBs and could advance precise multiplex editing of different genes (37, 114). But it also challenges the currently established tools for safety assessments as they mostly rely on the detection of unintended OT effects by DSBs (114). Unlike nuclease-based editing, BEs can introduce OT effects through multiple mechanisms, including gRNA-dependent DNA editing, gRNA-independent deamination of nucleotides, and OT RNA editing (115). Available assays such as Digenome-Seq and EndoV-Seq can detect base editor off-targets, but their reliance on whole-genome sequencing (WGS) (30–40x read depth) limits sensitivity to events above ~2–3% (116–119). Currently, safety assessments for base editing OT analysis include WGS with deep coverage to identify unintended single-nucleotide variants, as well as targeted deep sequencing of in silico predicted OT sites (**Figure 1**).

Seeking to identify further cell specific alternatives, RNA sequencing (RNA-seq) has been employed to detect OT RNA edits caused by some BEs. However, this approach has limited sensitivity for low-frequency edits and requires high read depth to detect subtle transcriptome-wide changes. To our knowledge they have not be applied in NK cells yet (120).

Finally, the recent advances in artificial intelligence (AI) and machine learning (ML) have the potential also to be implemented as forecast tools for advanced gene editing tools (121). Currently, they are most frequently used to improve gRNA design and OT predictions by scoring algorithms and ML models and recently as CRISPR-GPT, as an agentic for automation of gene editing experiments and data analaysis (121, 122). Today, one limitation for AI and ML models for OT detection, is the limited availability and small size of datasets for training validated deep learning models. However, the recent developments in the field will potentially overcome such limitations and can make AI and ML a promising tool for prediction of OT and chromosomal changes in the future.

Overall, the increasing frequency of CRISPR-editing in primary cells and its promising application for precisely designing and enhancing the inherent killing capacity of NK cells highlight the need for rigorous and comprehensive safety assessments. In addition to molecular and computational approaches, *in vivo* studies are gaining importance for providing a more complete picture of safety. They allow researchers to observe the persistence and distribution of engineered NK cells, as well as possible cytokine-related or off-target effects under more physiological conditions. Combining these *in vivo* insights with genomic analyses and the development of artificial prediction models, it can provide a clear comprehensive and precise framework for assessing the next-generation of safe off-the-shelf NK-cell treatments. Although the use of such tools is still scarce in NK cells, there is a strong need to apply and further develop these approaches in this emerging immune cell type to avoid compromising the highly promising safety profile of these cells. The parallel developments of multiplex editing and rigorous safety assessments tools will pave the way for the strong clinical translation of safe and effective NK cell products in the future.
